# Bridging the gap between complexity science and clinical practice by formalizing idiographic theories: a computational model of functional analysis

**DOI:** 10.1186/s12916-020-01558-1

**Published:** 2020-04-08

**Authors:** Julian Burger, Date C. van der Veen, Donald J. Robinaugh, Rick Quax, Harriëtte Riese, Robert A. Schoevers, Sacha Epskamp

**Affiliations:** 1 University of Groningen, University Medical Center Groningen, University Center Psychiatry (UCP) Interdisciplinary Center Psychopathology and Emotion Regulation (ICPE), Hanzeplein 1, 9713 GZ Groningen, The Netherlands; 2 University of Amsterdam, Institute for Advanced Study, Amsterdam, The Netherlands; 3 Harvard University, Department of Psychiatry, Massachusetts General Hospital, .Cambridge, MA USA

**Keywords:** Dynamical systems, Functional analysis, Computational modeling, Network analysis, Complex systems, Ordinary differential equations, Formalizing theories, Idiographic approach, Process-based psychotherapy, Theory development

## Abstract

**Background:**

The past decades of research have seen an increase in statistical tools to explore the complex dynamics of mental health from patient data, yet the application of these tools in clinical practice remains uncommon. This is surprising, given that clinical reasoning, e.g., case conceptualizations, largely coincides with the dynamical system approach. We argue that the gap between statistical tools and clinical practice can partly be explained by the fact that current estimation techniques disregard theoretical and practical considerations relevant to psychotherapy. To address this issue, we propose that case conceptualizations should be formalized. We illustrate this approach by introducing a computational model of *functional analysis*, a framework commonly used by practitioners to formulate case conceptualizations and design patient-tailored treatment.

**Methods:**

We outline the general approach of formalizing idiographic theories, drawing on the example of a functional analysis for a patient suffering from panic disorder. We specified the system using a series of differential equations and simulated different scenarios; first, we simulated data without intervening in the system to examine the effects of avoidant coping on the development of panic symptomatic*.* Second, we formalized two interventions commonly used in cognitive behavioral therapy (CBT; *exposure* and *cognitive reappraisal*) and subsequently simulated their effects on the system.

**Results:**

The first simulation showed that the specified system could recover several aspects of the phenomenon (panic disorder), however, also showed some incongruency with the nature of panic attacks (e.g., rapid decreases were not observed). The second simulation study illustrated differential effects of CBT interventions for this patient. All tested interventions could decrease panic levels in the system.

**Conclusions:**

Formalizing idiographic theories is promising in bridging the gap between complexity science and clinical practice and can help foster more rigorous scientific practices in psychotherapy, through enhancing theory development. More precise case conceptualizations could potentially improve intervention planning and treatment outcomes. We discuss applications in psychotherapy and future directions, amongst others barriers for systematic theory evaluation and extending the framework to incorporate interactions between individual systems, relevant for modeling social learning processes. With this report, we hope to stimulate future efforts in formalizing clinical frameworks.

**Electronic supplementary material:**

The online version of this article (10.1186/s12916-020-01558-1) contains supplementary material, which is available to authorized users.

## Background

Complex system thinking is gaining increasing importance in understanding mental health [1–3]. In recent years, some clinicians have proposed a move away from the approach of treating mental illness as disorder categories towards a focus on processes and patient-specific mechanisms in psychotherapy [4]. These proposals call for a framework for thinking about mental illness in terms of *systems*, to understand the *processes* underlying psychopathology, and to apply this understanding to *patient-specific* contexts. The network perspective to psychopathology [5–8], conceptualizing psychological disorders as complex interactions of symptoms and related mental health factors, provides a framework to address this movement. Statistical procedures that allow for the estimation of psychopathological networks have been developed [9–11] and applied across a wide range of mental disorders [12–15].

Furthermore, and arguably most relevant for psychotherapy, tools for *idiographic* network analysis have been developed [16, 17], allowing us to explore patient-specific symptom dynamics from data collected using the experience sampling method (ESM) [18]. This approach may be especially relevant for psychotherapy, as it has the potential to be embedded within clinical practice through informing the formulation of idiographic theories (i.e., case conceptualizations) and the identification of patient-tailored intervention targets [19]. Indeed, idiographic network analysis aligns well with the movement towards process-based psychotherapy [4]. It therefore seems surprising that, despite the availability of supportive statistical tools and efforts to provide primers for conducting idiographic research [20], the actual application of personalized network modeling within psychotherapy is to date rare.

### From implementation barriers to a clinician’s wishlist

Implementation gaps between mental health research and clinical practice are a topic of enormous importance [21, 22]. With the emergence of the complex system approach in mental health research, there has been specific interest in implementing statistical tools to explore patient-specific symptom dynamics in clinical practice. It is commonly assumed that successful implementation is in part a question of providing technical trainings and accessible guidelines for clinicians [20]. However, merely training clinicians in adopting tools provided by methodologists does not guarantee that these tools also result in models that map onto the *language* used by practitioners. Indeed, an often-discussed barrier to implementation is the accurate *translation of knowledge* into the relevant practice field [21]. That is, the language used to discuss promising research findings and techniques does not always match the targeted language of the practitioner.

This issue applies to the estimation of personalized network models. At present, network estimation methods remain technical and do not account for potentially relevant clinical considerations. For example, network estimation methods identify “highly central” symptoms, given some assumptions, as promising targets of intervention [19], but these methods generally fail to account for the fact that symptoms differ in their amenability to psychological treatment or that some symptoms may have “low centrality” but remain critical targets for intervention because of their impact on psychosocial functioning (e.g., suicidal thoughts and behavior; [22, 23]). Further, currently available techniques to estimating personalized networks are primarily of *exploratory* nature and do not allow clinicians to incorporate relevant a priori knowledge or clinical expertise. By failing to see their ideas reflected in network models, practitioners might consider them as impractical and not in line with their clinical view, likely resulting in hesitancy towards using personalized network models. Indeed, a recent study has shown that case conceptualizations greatly differ from temporal networks estimated from ESM data [24].

Based on these considerations, we argue that providing trainings and guidelines is necessary, but not sufficient in implementing the complex system approach in clinical practice. For methods to be regarded clinically relevant, it is vital that tools have the flexibility to be guided by clinical needs and allow practitioners to incorporate clinical considerations.

### Theories versus data models

In recent literature, special attention has been paid to disentangling conceptual aspects of *data models* and *theories*. According to Haslbeck, Ryan, Robinaugh, Waldorp, and Borsboom [25], data models (e.g., a mean, correlation, or idiographic network model) are merely ways of representing or organizing data, often with the aim of establishing a phenomenon: a robust, generalizable feature of the world identified through empirical regularities [25, 26]. In contrast, the aim of a theory is to explain a phenomenon by representing those aspects of the real world that give rise to the phenomenon. Whereas verbal theories are expressed in language, formal theories are expressed in mathematical equations or a computational programming language. This level of specification allows formal theories to simulate theory-implied system behavior, and by observing the effects of simulated interventions, we can draw conclusions about how the real-world system we are targeting would respond to a given treatment (a process referred to as “surrogative reasoning”, cf. [27]).

In the following, we will refer to the approach of translating (verbal) case conceptualizations into mathematical systems as the *formalization of idiographic theories*. Although the term “theory” is commonly used to describe phenomena on the nomothetic level, in this paper, we are focused on the explaining phenomena at the level of the individual patient, and will use the term “idiographic theory” in respect to theorized relations within one individual.

### Formalizing idiographic theories

To bridge the gap between methodological advances and practical application of the complex system approach, we propose to derive dynamical system models directly from clinical theory, clinicians’ expertise and case-specific knowledge. Formalizing patient systems tackles the mismatch between technical tools and target language as discussed above at its core; that is, rooting dynamical systems *in the language of practitioners* allows examining the patient’s system behavior based on clinically relevant considerations.

In other scientific disciplines like biology [28], ecology [29], and political science [30], it is common to model dynamic processes based on theory and/or knowledge. Unfortunately, the application of formalized theories in mental health research is to date extremely rare. Recently, there have been efforts to propose formal theories in psychiatry, including the relationship between patient and therapist [31] and models of burnout [32, 33], addiction [34], and panic disorder [35]. However, much remains unknown about precisely how such formal theories should be developed and how they should be used in psychotherapy. The main objective of this paper is to take a step towards addressing this gap in the literature by demonstrating the potential of formalizing idiographic theories in clinical practice and illustrating an approach to formalizing such theories using the framework of functional analysis.

### Approaches to constructing idiographic systems

We see two main ways of constructing personalized dynamical systems in psychopathology: First, modeling a *generic* disorder model, and subsequently personalizing the model through estimating control parameters for the equations in the system (*top-down approach*, cf. [35]), and second, modeling relations between *specific* variables directly for and with each patient (*bottom-up approach,* cf. [36–39]). An advantage of the former approach is that it allows modeling individual differences between patients regarding the strength of *shared* relations (e.g., person-specific tendencies to avoid when confronted with fear), which consequently allows for examining for instance tipping points in fear responses following maladaptive coping. An advantage of the latter approach is that it allows to flexibly model any psychological hypotheses, as well as individual problems and resources [40].

The method outlined in this paper is based on the framework of functional analysis, and therefore utilizes elements of both approaches: On the one hand, functional analysis constitutes a generic framework for case formulation (*top-down* elements); on the other hand, it also provides the flexibility to integrate patient-specific problems and resources (*bottom-up* elements).

### The role of computational models in bridging the scientist-practitioner gap

**Fig. 1:**
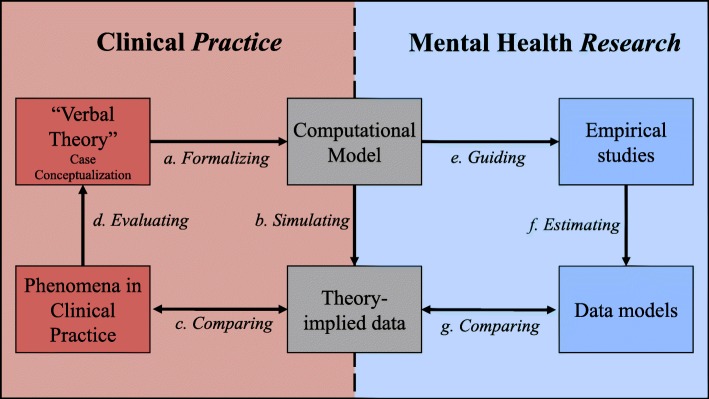
The role of computational modeling in bridging the scientist-practitioner gap. Schematic illustration of computational modeling (the product of formalizing a theory), at the intersection of clinical practice and mental health research. Computational models allow us to evaluate case conceptualizations in clinical practice (a–d), and bring clinical theories closer to empirical studies through guiding choices crucial to the estimation of and inferences drawn from data models (b, e–g)

We argue that formalizing idiographic theories provides advantages for both, clinical *practice* and mental health *research*, schematically displayed in Fig. 1, and is promising in bridging the gap between the two.

First, computational models of idiographic theories can be used to advance the current practice of a patient’s case conceptualization. Sim, Gwee, and Bateman [40] identified five key advantages associated with formulating thorough case conceptualizations in clinical practice: (a) the integration/relation of multiple problems of a patient, (b) the explanatory nature of the resulting model, (c) the prescription of interventions, (d) the prediction of outcomes, and (e) the support for the therapeutic relationship. Schiepek and colleagues [36, 37, 41] pioneered the integration of case formulation and idiographic system modeling and argued that these key advantages could be strengthened through computational models. Clinicians are required to make more rigorous decisions in specifying relations in the case conceptualization, which makes the formalization of idiographic theories a promising avenue to foster more scientific practices in designing patient-tailored treatment. This reasoning is in line with a growing body of literature indicating the need for more rigorous theory development in clinical and social sciences [25, 35, 42–45]. The left part of Fig. 1 illustrates how computational modeling can inform case conceptualizations in clinical practice: Formalizing a case conceptualization results in a computational model that allows the clinician to subsequently simulate data, given the specified idiographic system. Based on these simulations, it is possible to compare theoretical implications to phenomena observed in clinical practice and to evaluate and adapt theory accordingly [25, 46, 47]. Theory formation can thus be adapted by examining *what a theory implies*, and these implications only become fully apparent once a theory is formalized and data can be simulated.

Second, computational models bring clinical theories closer to empirical *research.* For instance, prior to empirically studying a patient’s systems, the researcher needs to determine variables to include into the analysis. This question is of great importance in network estimation, since parameters in partial correlation networks are heavily dependent on the set-up of variables. The choice of variables has a crucial impact on network estimation and inference, especially if clinically relevant variables are missing, or if included variables stem from theoretically similar constructs, indicating topological overlap [48]. Formalizing theories can provide useful information regarding the set-up of variables needed to retrieve clinical phenomena. Further, empirical research is often confronted with practical constraints to assessing psychological processes. Many clinically relevant psychological processes are difficult—sometimes even impossible—to assess on their appropriate time-scale. For practical reasons, variables are often measured within the same time-scale (usually once a day or every few hours), potentially leading to biased estimates in dynamical models. A recent simulation study suggests that using the most commonly applied ESM time-intervals results in data models that are largely unable to recover the micro dynamics of a system [49]. A stronger focus on theory and the utilization of clinical knowledge could therefore be helpful in informing relationships in the estimated model that cannot reasonably be captured by commonly used ESM data. The right part of Fig. 1 illustrates how computational modeling can guide mental health research, resulting in data models that are grounded in theory-based considerations. The resulting data models can be compared against theory-implied simulation results and guide further theory development [25] as well as future research design planning.

### Example of a computational model: functional analysis of patient with panic disorder

In the remainder of this paper, we will introduce and evaluate an example system based on *functional analysis* (sometimes referred to as *applied behavior analysis* or *SORKC model* [50]), a framework commonly used by clinicians to formulate case conceptualizations in CBT. Functional analysis explains maladaptive behavior in terms of classical and operant conditioning processes: a discriminant stimulus (*Sd*) evokes specific emotional, cognitive and behavioral responses in the patient (*Re*, *Rc*, and *Rb*, respectively). Persistent dysfunctional coping is explained through the presence of reinforcing stimuli. In the short term, dysfunctional coping mostly yields positive effects (*perceived benefits*), while on the long term, negative effects (*perceived costs*) are accumulating.

To illustrate, we are modeling the case conceptualization of a hypothetical patient suffering from panic disorder. This example patient experiences unusual bodily sensations (arousal) in the cinema and concludes that she will have a heart attack and that there is no chance she can get medical assistance on time. The experience of heart racing in the cinema constitutes her *discriminant stimulus* (*Sd*). Her emotional response is panic (*Re*), due to catastrophic interpretations of the heart racing (“I am having a heart-attack”; cognitive response, *Rc*). In order to cope with the aversiveness of this situation, she leaves the cinema (*Rb*). This behavior yields benefits: The patient manages to decrease the intense fear she felt in the cinema (*perceived benefits*). However, constant avoidance also leads to costs: The patient withdraws herself socially and experiences problems at work due to her avoidant coping in panic-evoking situations (*perceived costs*). Further, she is faced with a lack of falsification possibilities, increasing the *credibility of her catastrophic thoughts* in confrontation with experiencing heart racing while not being able to get medical assistance.

**Fig. 2:**
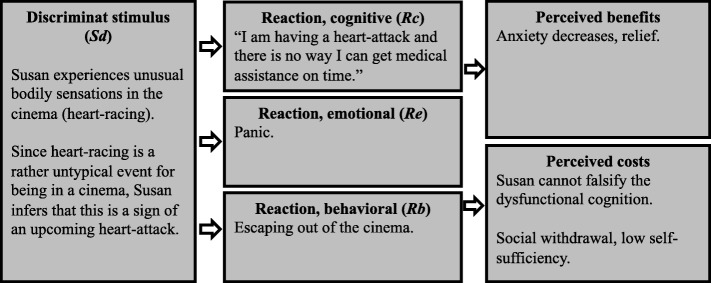
Functional analysis of hypothetical patient suffering from panic disorder. Case conceptualization of our example patient using the framework of functional analysis, as commonly documented in clinical practice. A discriminant stimulus leads to cognitive, emotional, and behavioral reactions (*Rc*, *Re*, *Rb*, respectively). The behavioral reaction has perceived benefits and costs, reinforcing or inhibiting the behavior

Figure 2 shows a schematic summary of the main factors involved in the patient’s functional analysis, as typically documented in psychotherapy. Robinaugh and colleagues recently proposed a computational model for panic disorder [35]. While Robinaugh et al. focus on the generic approach described above, we also include personal factors as components in the model, in accordance with the principles of functional analysis. As will be discussed later on, patient-specific reinforcing factors can be modeled through both, extending equations and altering parameters in the system.

## Methods

In the following, we describe the general approach to formalizing idiographic theories, using the functional analysis of our hypothetical patient. To facilitate readability, we focus on introducing the process on a *conceptual* basis. We advise the reader interested in technical detail to consider the supplementary material (see Additional file 1: Mathematical Background). Note that the simulation results and the discussion can be followed without having read the mathematical background section.

In many formal theories, including the one that will be presented here, every component of the system is expressed as a *differential equation*, precisely explicating the specific influences of system variables on one another. Intuitively, differential equations can be understood as specifying the rate of change in a given variable (i.e., how a given variable will change over time), as a function of itself and other causally related variables. For instance, in the simplest case of a first-order derivative, the differential equation of the variable *avoidance* captures the extent to which avoidance behavior will increase or decrease moving forward from a given time point. Since our system predicts that avoidance is employed as a consequence of anxiety, the corresponding differential equation would encode that high levels of anxiety increase the first-order derivative (the momentary change) of avoidance.

Note that in this paper, we primarily focus on modeling *linear* differential equations. Extending the framework to including *non-linear* equations would be a relevant step for future research, given that prior literature found that psychotherapeutic processes are often chaotic, a feature that is characteristic for non-linear dynamics [41, 51]. For the sake of implementation, however, we decided to focus on linear equations, since many aspects of non-linear dynamics require an extensive mathematical understanding. We will discuss the difference between these approaches and the impact on predictions in systems later on.

### Procedure of formalizing idiographic theories

#### Step 1: Schematic representation

**Fig. 3:**
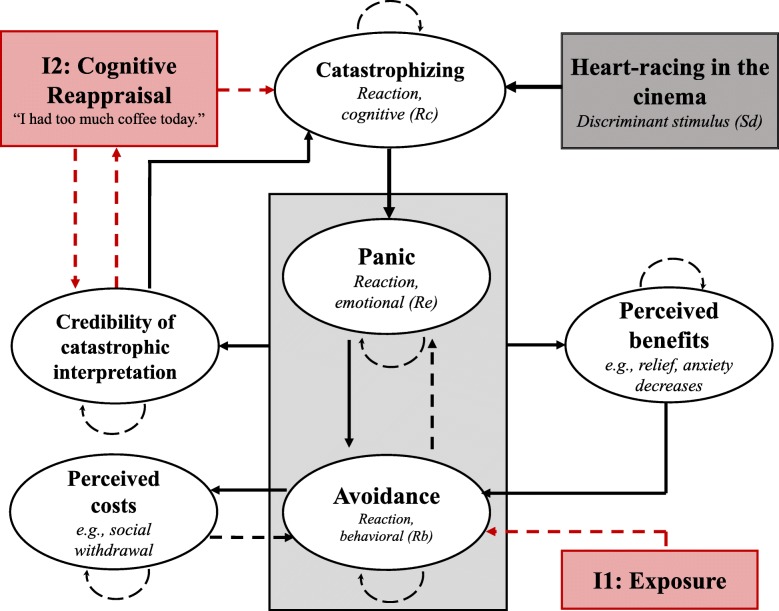
Schematic representation of the functional analysis. Theoretical relations adapted from the patient’s functional analysis as a basis for deriving the system equations. Anxiety (*Re*) is reduced through applying avoidance behavior (*Rb*). In addition, avoidance behavior is reinforced through *perceived benefits* and inhibited through *perceived costs*. Persistent avoidant behavior increases the *credibility of catastrophic interpretations*, in turn leading to more *catastrophizing* during exposure. We formalized and tested three interventions, *exposure*, *cognitive reappraisal*, and their combination, represented through the red boxes

Prior to formulating differential equations, we recommend visualizing the system schematically. This facilitates specifying relations in the equations later on. A graphical depiction of the relations in the patient’s functional analysis, including the target nodes of the interventions introduced below, is presented in Fig. 3. This is a crucial step, since it opens the search horizon beyond the given boundaries of functional analysis (i.e., allowing to incorporate person-specific elements into the system, such as competencies and resources), and requires the clinician to explicate relations between the variables.

#### Step 2: Deriving differential equations

Based on the schematic representation of the patient’s functional analysis, we formulated differential equations for each component in the system. Practical guidelines for defining dynamical systems from both theory and data have been recently described elsewhere [52].

As a starting point, we modeled catastrophic interpretations (*Rc*) of the discriminant stimulus (*Sd*) as input for the occurrence of panic (*Re*); heart racing in the cinema leads to the catastrophic idea that this is a sign of an upcoming heart attack, and the patient consequently experiences panic symptoms. In turn, the patient copes through avoidance behavior. We modeled coping behavior using equations commonly applied to model the dynamics between prey and predator populations in ecology [53]. In our model, panic (*Re*) is analogous to “prey” and avoidance (*Rb*) is analogous to “predator”. Thus, increases in panic give rise to increases in avoidance behavior, while increases in avoidance behavior lead to lower panic.

Avoidant coping is modulated through the presence of reinforcing/inhibiting factors. First, if the patient perceives avoidance to be effective in decreasing panic (i.e., experiencing relief; *perceived benefits*), her tendency to cope through avoidance increases. Second, avoidance behavior comes with detriments for the patient, for instance social withdrawal or potential problems at work. These detriments (*perceived costs*) are theorized to have an inhibiting effect on the patient’s avoidance behavior. Third, persistent application of avoidance behavior comes with a lack of opportunities to falsify the catastrophic interpretation. Therefore, we modeled increasing *credibility of the catastrophic interpretation* as a consequence of avoidant coping. The credibility of the catastrophic interpretation increases the patient’s tendency to catastrophize in confrontation with the discriminant stimulus.

#### Step 3: Formalizing interventions

One of the main advantages of computational modeling in clinical practice is that interventions on a system can be examined in silico, and their effects evaluated on the basis of a case conceptualization. Note that the simulated effects are dependent on the accuracy of the model, highlighting the importance of theory evaluation [25]. We will discuss future avenues for systematic evaluations later on.

Similar to step 2, interventions need to be formalized. We modeled two commonly used interventions in CBT: *exposure therapy* and *cognitive reappraisal*. First, we implemented exposure through setting avoidant coping to 0. Second, cognitive reappraisal was implemented through formalizing another system variable, capturing the credibility of an alternative *functional* interpretation of heart racing. The credibility of the functional interpretation was theorized to “compete” with the credibility of the catastrophic interpretation, and we thus formalized the former as an inverse function of the latter; if the *functional* interpretation of the stimulus increases, the *dysfunctional* interpretation decreases and vice versa. This change in interpretation of the stimulus influences the extent to which the patient catastrophizes. We therefore extended the equation for catastrophizing with an inhibitive term; increasing the credibility of the functional interpretation (e.g., “I simply had too much coffee”) leads to less catastrophic interpretations of the discriminant stimulus.

#### Step 4: Choosing initial values of system variables and parameters

Prior to conducting simulations, initial values of each system variable and parameters need to be defined. In contrast to many data-driven approaches of estimating networks, these values are difficult to interpret numerically. This is because formalizing idiographic theories does not require the clinician to *operationalize* variables, since these will not (necessarily) be *measured*. The units of system variables are therefore not meaningful. We will discuss advantages and disadvantages of aligning theory components with the measurement procedure later on.

In contrast to common parameter estimation techniques in data models, the approach outlined in this paper treats parameters in formalized theories as “tuning-knobs” to tailor the relations towards the patient’s case until theory-implied behavior resembles phenomena of interest. For instance, one can increase the parameter encoding the extent to which avoidance behavior follows panic, if it is known that the patient has a strong tendency to employ avoidance behavior as coping. Further, one can vary values of parameters to examine differential effects of *unknown* relations; for instance, clinician and patient can collaboratively examine the effects of different parameter choices for catastrophizing leading to panic. This allows patients to experimentally examine the responses of their system towards alterations.

For our example model, we chose parameters and initial values of the variables according to a qualitative examination of the system behavior, i.e., through adjusting parameters until the system resembled behavior to be expected given the information on the case of our hypothetical patient. The choice of parameters and initial values can be found in the mathematical appendix (see Additional file 1: Mathematical Background), alongside all differential equations used in the simulations.

#### Step 5: Simulating and visualizing theory-implied data

Following the system specification, we can simulate and visualize data. We provide the code to reproduce our analysis and plots in R (Additional file 2: Code to reproduce analyses). System data is commonly visualized in time-series plots and phase portraits. Time-series plots indicate the time trajectories of all system variables, with time on the *x*-axis and variable levels on the *y*-axis. Phase portraits are useful to display the relationship between two or three variables over time. Each variable is represented on an axis, and following the trajectory in the phase portrait gives us information regarding the time course of the displayed variables. To illustrate, we used the example of three-dimensional phase portraits, indicating the relationship between *panic*, *avoidant coping*, and *the credibility of the catastrophic interpretation*.

#### Step 6: Evaluating case conceptualizations

In a last step, the simulated (“theory-implied”) data can be compared to phenomena observed in clinical practice. Differences between simulated data and observed patterns can be an indication that specific system relations need to be adapted or that important variables are missing in the system [46, 47]. As illustrated in Fig. 1, these considerations can be important pointers for setting up empirical investigations of symptom dynamics (e.g., which variables to include in an ESM study). We will address formal aspects of theory evaluation in the “Discussion” section.

## Results

### Scenario 1: System behavior without intervention

**Fig. 4:**
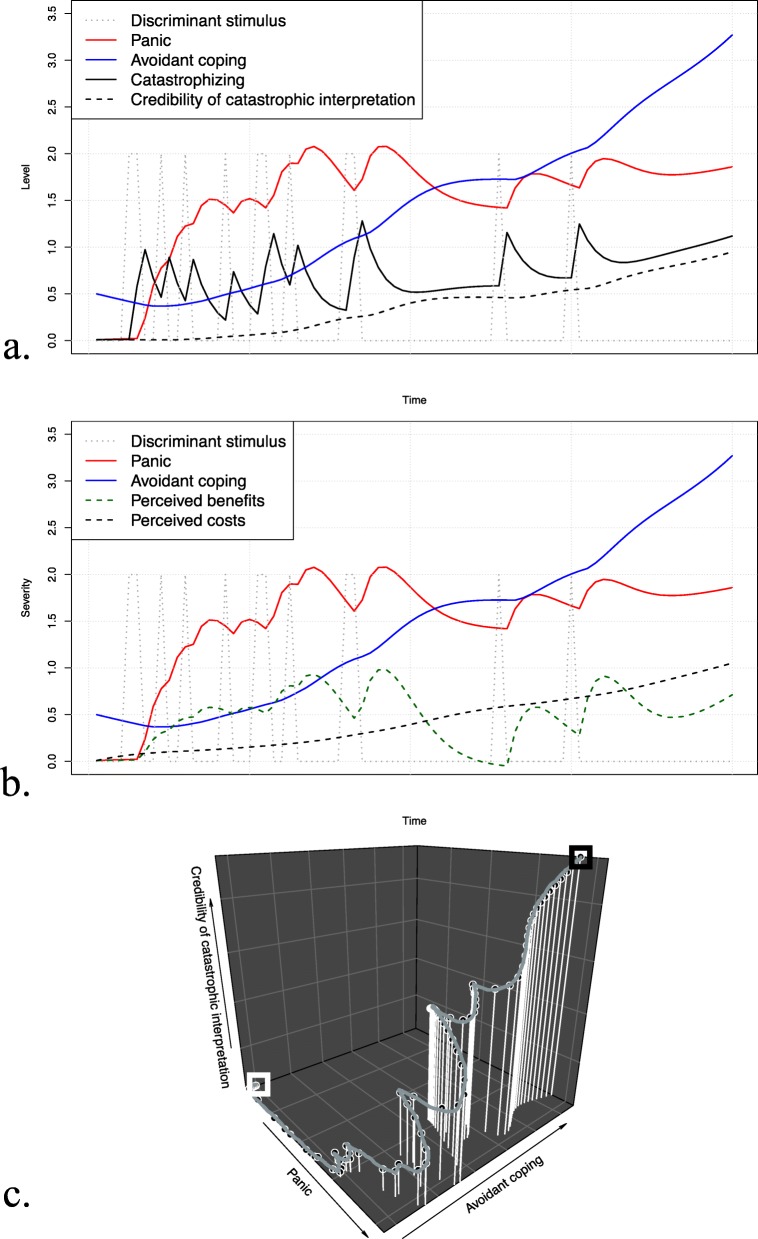
Simulation results of scenario 1 (no intervention). The top and middle parts show the simulated time-series for the discriminant stimulus, panic, and avoidant coping along with **a** catastrophizing and the credibility of the catastrophic interpretation and **b** perceived benefits and costs. The bottom part of the figure (**c**) shows the three-dimensional phase portrait for panic, avoidant coping, and the credibility of the catastrophic interpretation, where the white box indicates the start and the black box the end of the trajectory

Figure 4 a–c show time-series plots and phase portraits for the simulated system behavior without intervention. Being confronted with the discriminant stimulus led to a rapid increase in catastrophizing, followed by panic. Over time, avoidance behavior gradually built up as a coping mechanism. While this was associated with a momentary decrease in panic, persistent avoidance was also accompanied by increasing credibility of the catastrophic interpretation, in turn leading the patient to catastrophize even more when confronted with the discriminant stimulus. In the short term, avoidance behavior was mainly associated with benefits, while in the long term, the perceived costs built up. The three-dimensional phase portrait shows that persistent avoidance behavior did not allow the patient to decrease panic states in the long term. Instead, panic tendencies manifested as a function of the credibility of the catastrophic interpretation. A clinical interpretation could be that the patient was not able to falsify catastrophic interpretations due to the lack of exposure to the discriminant stimulus.

### Scenario 2: Behavioral therapy (*exposure*)

**Fig. 5:**
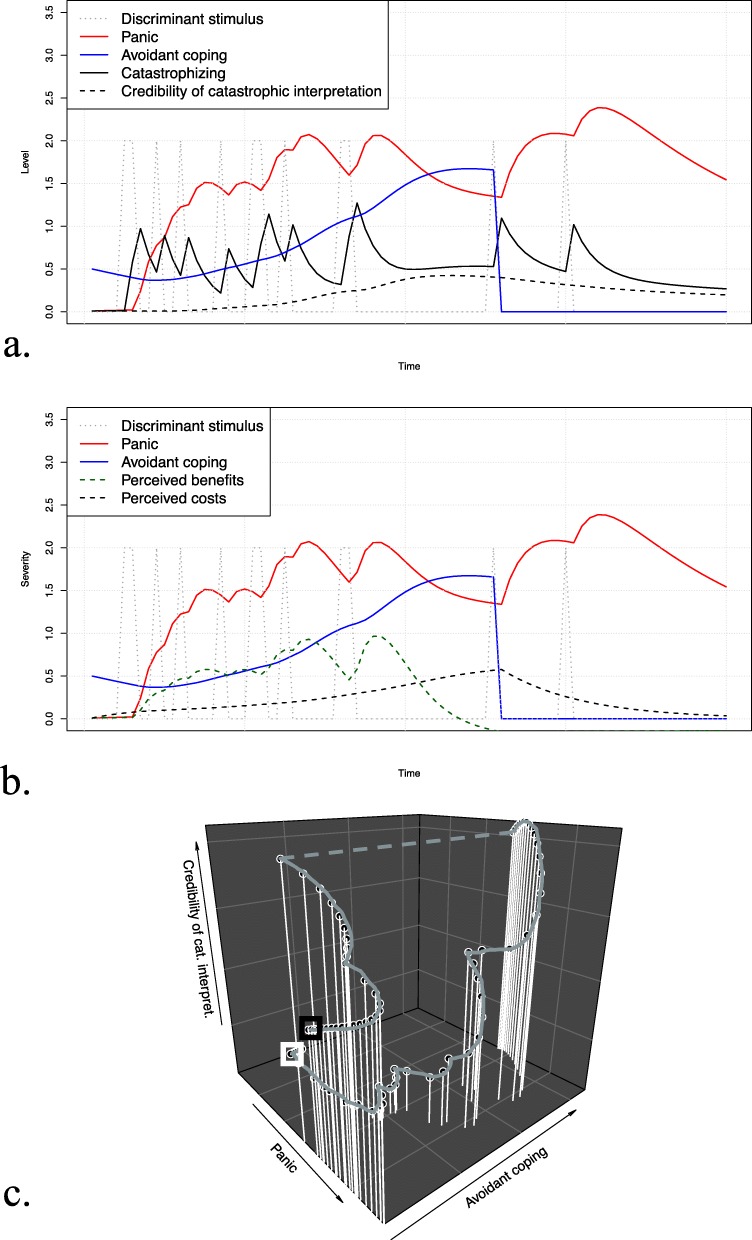
Simulation results of scenario 2 (exposure; behavioral therapy). The top and middle part show the simulated time-series for the discriminant stimulus, panic, and avoidant coping along with catastrophizing and the credibility of the catastrophic interpretation (**a**) and perceived benefits and costs (**b**). The bottom part of the figure (**c**) shows the three-dimensional phase portrait for panic, avoidant coping, and the credibility of the catastrophic interpretation, where the white box indicates the start and the black box the end of the trajectory

Figure 5 a–c show the time-series plots and phase portrait when applying exposure. This intervention led to a sudden increase in panic states in the short term. In the long term, panic decayed even under absence of avoidant coping, accompanied by a decrease in catastrophizing and credibility of the catastrophic interpretation, demonstrating the effectiveness of behavioral therapy for our patient. With the introduction of exposure therapy, the perceived benefits of avoidance behavior disappeared, e.g., the patient could not experience relief through avoidance anymore, and the associated costs decayed over time.

### Scenario 3: Cognitive therapy (*cognitive reappraisal*)

**Fig. 6:**
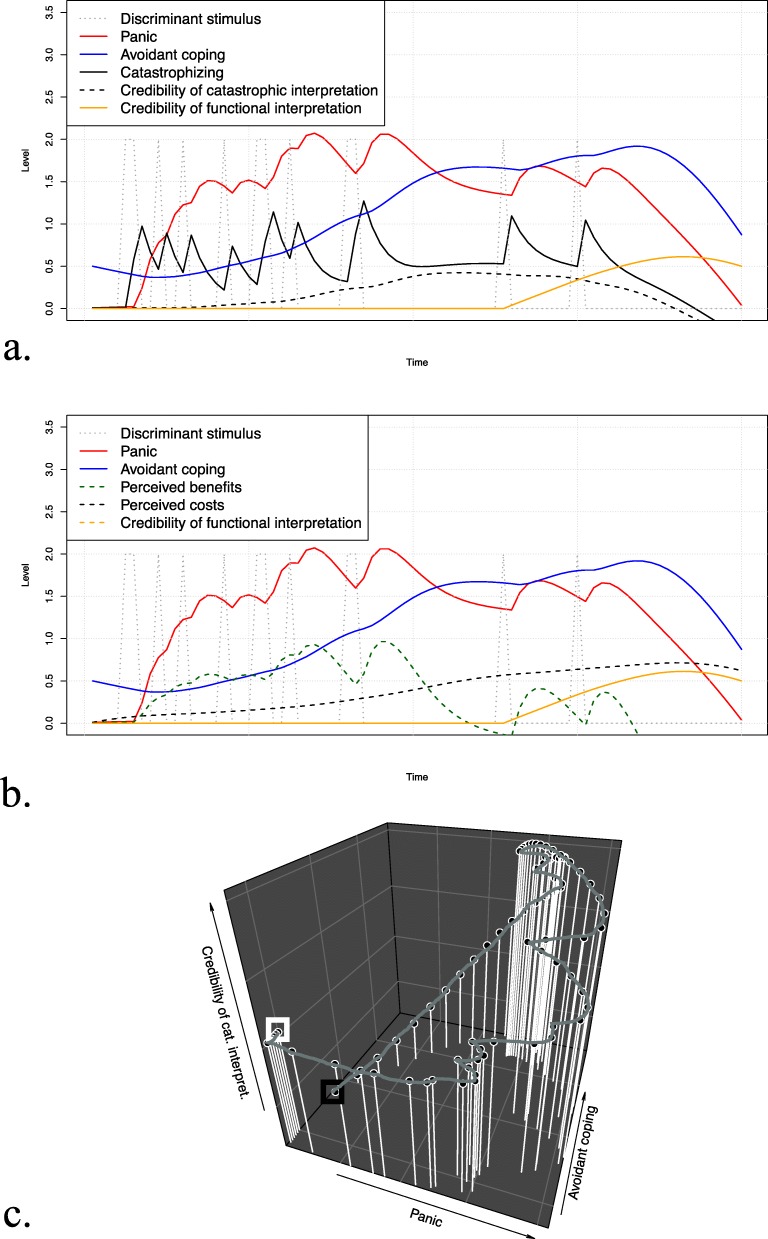
Simulation results of scenario 3 (cognitive reappraisal; cognitive therapy). The top and middle part show the simulated time-series for the discriminant stimulus, panic, and avoidant coping along with catastrophizing and the credibility of the catastrophic interpretation (**a**) and perceived benefits and costs (**b**). The bottom part of the figure (**c**) shows the three-dimensional phase portrait for panic, avoidant coping, and the credibility of the catastrophic interpretation, where the white box indicates the start and the black box the end of the trajectory

Figure 6 a–c show the time-series plots and phase portrait when applying cognitive reappraisal. While functional interpretations of the discriminant stimulus could help decreasing panic tendencies, avoidance behavior only decreased after the functional interpretation gained sufficient credibility. Additionally, catastrophizing and the credibility of the dysfunctional cognition decreased, while avoidance behavior gave rise to both, the perceived costs and benefits.

### Scenario 4: Cognitive behavioral therapy (*exposure* + *cognitive reappraisal*)

**Fig. 7:**
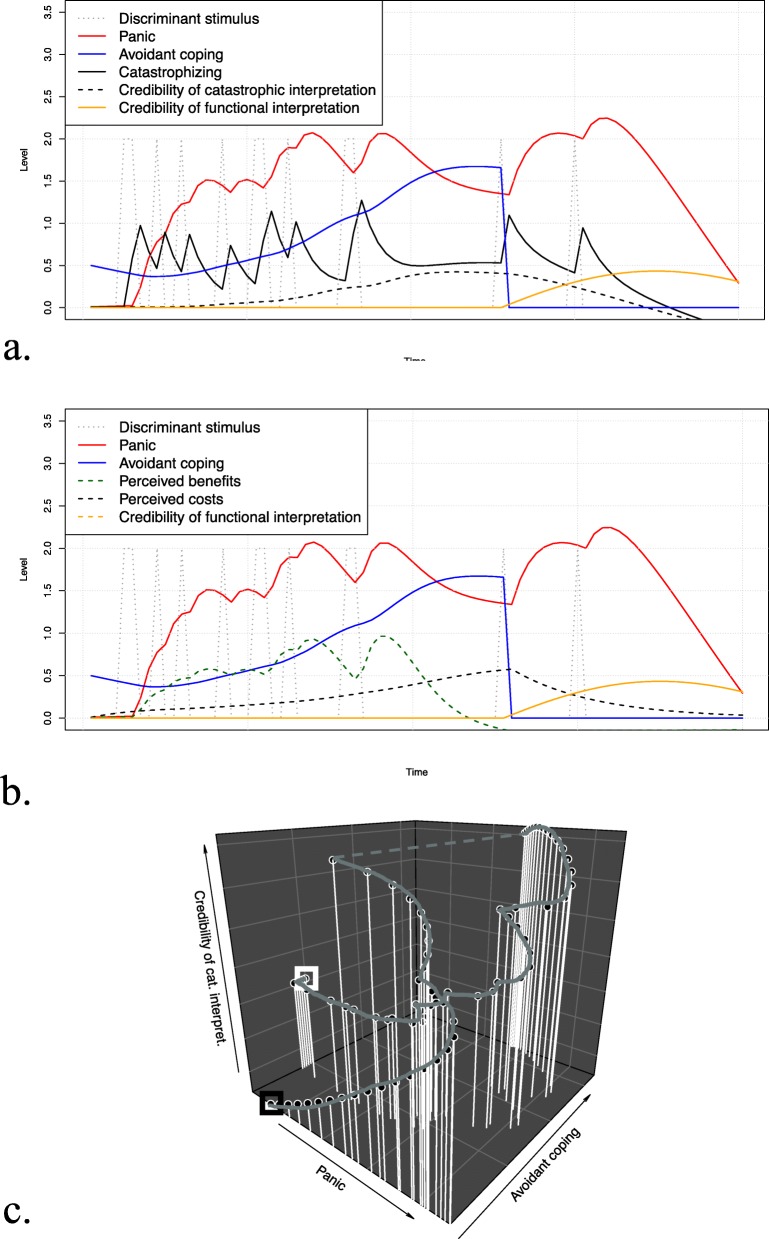
Simulation results of scenario 4 (exposure and cognitive reappraisal; CBT). The top and middle parts show the simulated time-series for the discriminant stimulus, panic, and avoidant coping along with catastrophizing and the credibility of the catastrophic interpretation (**a**) and perceived benefits and costs (**b**). The bottom part of the figure (**c**) shows the three-dimensional phase portrait for panic, avoidant coping, and the credibility of the catastrophic interpretation, where the white box indicates the start and the black box the end of the trajectory

Figure 7 a–c show the time-series plots and phase portrait when applying exposure and cognitive reappraisal simultaneously. Similar to scenario 2, this combination of interventions led to an increase in panic tendencies in the short term. The introduction of the functional interpretation of the discriminant stimulus was accompanied by a decrease in catastrophic interpretation and its credibility, ultimately leading to a decrease in panic tendencies. Similar to scenario 2, confrontation led the associated benefits of the behavior to disappear and the costs to decay over time.

## Discussion

Current movements in psychotherapy strongly align with technical advances in dynamical modeling tools—yet their implementation in clinical practice is rather scarce. To bridge this gap, we call for a stronger focus on tools that make use of *frameworks and theories* embedded in clinical practice. In this paper, we discussed the formalization of idiographic theories, through the use of differential equations, as an alternative to data-driven network modeling approaches. Our main objective for promoting the use of formalized idiographic theories is that data models cannot always account for considerations relevant to clinical practice. In consequence, even though techniques seem to be promising in analyzing patient data, their implementation might be hampered due to the lack of options to incorporate theoretical and practical considerations. This barrier can be addressed through grounding dynamical systems in the theories of practitioners. Differential equations are commonly used in a variety of other scientific fields to describe systems, and are a promising avenue for formalizing theories of mental health.

To illustrate this approach, we formulated a computational model based on dynamics of the functional analysis for a patient suffering from panic disorder and examined implications for the case conceptualization and the effects of commonly applied CBT interventions. The results of the simulations are largely congruent with phenomena observed in clinical practice and in line with predictions of other theoretical frameworks. In the following, we discuss further benefits for clinical practice, concrete examples for theory adaptation, and future directions.

### Benefits for clinical practice and clinical relevance

We identify at least five benefits from formalizing case conceptualizations in respect to challenges faced in clinical practice.

#### Scientific rigor

One of the main advances in mental health care over the past decades is its increasing focus on scientific practices. The introduction of the scientist-practitioner model [54] was an attempt to strengthen scientific practices in psychotherapy, for instance through theory-guided hypothesis testing. It became vital for designing patient-tailored psychotherapy to formulate a testable theory regarding intervention effects. The case conceptualization is an example of a framework for such scientific theories in clinical practice. However, if a theory is vague, the resulting hypotheses, predictions, and tests become scientifically questionable [45]. Especially in the current landscape of replicability issues [55, 56], we see value in enhancing theory development through formalizing idiographic systems in clinical practice. As became evident in this report, especially when comparing the initial *verbal* theory in Fig. 2 to the system of differential equations, the process of formalizing idiographic theories is mostly a process of increasing specificity, in which clinicians need to thoroughly reflect on and justify all relations between system variables.

#### Idiography

While the model used in this paper uses concepts that are relevant for a broader range of patients suffering from panic disorder (*generic approach*), there are many individual differences in how exactly these relations should be specified. For instance, patient A might have more exposure to their discriminant stimulus in their everyday life compared to patient B, or patient C has stronger avoidance tendencies than patient D. These considerations can be reflected in altering the parameters in the system, aligning this approach with the idea of idiographic modeling. Further, specific components in the system can be added/removed, if applicable for a given individual. The framework of functional analysis is transdiagnostic in nature and can be applied to a broad range of disorders that involve dysfunctional coping, for example, substance abuse, post-traumatic stress disorder, obsessive-compulsive disorder, and depression.

#### Explanation

Functional analysis provides a framework that allows explaining the function of maladaptive behavior and helps understanding symptom maintenance. The explanatory character of these verbal theories can be advanced through formalization, since case conceptualizations can subsequently be evaluated in respect to how well they can reproduce clinical phenomena [46, 47]. If a case conceptualization fails to explain relevant phenomena, this will more easily be detected if data is simulated from a formalized case conceptualization, compared to a verbal theory.

#### Prediction

While computational modeling can foster the development of theoretical relations, it is also a useful tool for predicting theory-implied system behavior under given interventions. Most relevant for clinical practice, this allows the clinician to examine the effects of formalized clinical intervention in silico. Testing interventions in computational models offers efficient insight into intervention effects without having to collect data.

#### Didactics

Simulation outcomes of a formalized idiographic theory can be beneficial for didactics in clinical practice. First, visualizing the simulation results allows the clinician to collaboratively examine symptom dynamics with the patient. This can be used in the process of psychoeducation, and communicating a treatment rationale, especially for interventions that might be aversive for the patient (e.g., exposure). Second, in the long term, we see potential in implementing formalized idiographic theories to enhance more concise communication between clinicians through more rigorous documentation and visualization.

### Theory evaluation of the example model

A main benefit to formalizing idiographic theories is that simulated data can directly be compared against expected/reported behavior in the patient. One potential interpretation of discrepancies between simulated data and clinical phenomena is that the case conceptualization in its current form cannot account for potentially relevant clinical phenomena, for instance, if important relations or variables are missing. If this is the case, the clinician might want to adapt specific theoretical relations until the simulated data adequately represents clinical phenomena. This is crucial when testing formalized interventions in a patient’s system.

In some aspects, the computational model presented in this paper is congruent with clinical phenomena, while in other aspects theory adaptation might be needed. Note that the set-up of the simulation represents panic-symptomatology experienced by one hypothetical individual. Phenomena observed in simulations might differ if parameters are altered, which allows capturing individual differences in experiencing panic symptoms, and differences in treatment response. First, the simulations showed that for this patient, persistent avoidance behavior is accompanied by increasing tendencies to catastrophize and increasing credibility of the catastrophic interpretation. This finding highlights the role of falsification in fear disorders; avoidant coping is associated with a lack of opportunities to falsify the catastrophic interpretation, subsequently leading to increasing tendencies to experience panic in confrontation with discriminant stimuli. Second, the simulations indicate that all interventions (exposure, cognitive reappraisal, and combination) are effective in decreasing panic tendencies for this patient, which is in line with empirical studies testing the efficacy of CBT interventions for panic disorder [57]. Third, the simulation results showed that panic manifests in the long term, if no intervention is applied. This finding does not seem to adequately represent the experience of panic attacks, since these usually emerge rapidly and decline after a short amount of time. To account for this feature of panic attacks, we propose to model stronger decay of panic. Alternatively, one could conceptualize this variable as a *tendency* to experience panic in the presence of the discriminant stimulus, rather than the actual experience of panic itself.

### Future directions

The approach of formalizing idiographic theories is still fairly new to clinical psychology, and there is a lot of research that needs to be conducted to help implementing it in clinical practice. In this section, we aim to give some directions for future research.

#### Systematic theory evaluation and testing

A crucial barrier for implementation is that the explanations and predictions provided by a theory need to be as accurate as possible, especially if the aim is to test formalized clinical interventions; such interventions will depend heavily on the accuracy of the model. We outlined that through comparisons of theory-implied and empirical data, systems can be evaluated to increase accuracy. Notably, any *systematic* comparison between theory-implied and empirical data models would require that variables used in *data collection* either directly map on to components in the *theory*, or that they can be precisely derived from those components. As outlined above, there are many elements in idiographic systems that are difficult to capture in common forms of data collection (e.g., ESM data), suggesting direct mapping of theory components to variables in empirical data may be difficult. Accordingly, it will be necessary for researchers to not only formalize theories, but also the auxiliary hypotheses about measurement that link the theory components to the variables in empirical data. In this paper, we opted for modeling idiographic systems without restrictions to what can be operationalized and compared how well theory-implied data qualitatively resembles clinical phenomena based on expert discussions, but did not go through the process of formalizing our assumptions about measurement or deriving what should be expected in any given empirical data model.

Second, it needs to be noted that the origin of a potential mismatch between theory-implied and empirical data remains unknown. Such discrepancies can have a multitude of sources and can be ascribed to either shortcomings in the structure of the theory (e.g., missing crucial variables in the theory, mis-specified or missing relations between present elements of the theory), the set-up of the simulation (e.g., exact initial conditions, valid parameter values, input and boundary conditions), or shortcomings in empirical data collection and modeling (e.g., inappropriate modeling assumptions, measurement issues). Further, estimating parameters from non-linear time-series data is often difficult and undergoes strong limitations [58]. We call for future research to investigate systematic ways of identifying the core of such discrepancies.

#### Technical expertise and effort

Another barrier to implementation is that, in the current practice of formalizing idiographic theories, constructing a series of differential equations to formalize a patient’s system can be immensely challenging and requires technical expertise that is not part of psychotherapy trainings. To address this issue, we propose that methodologists elaborate on a set of functions relevant to relations between clinical variables that can readily be used by clinicians to formalize idiographic theories. To enhance accessibility, this set of functions could be implemented in an interactive tool to visualize variable interactions. Clinicians could then pick from this set and construct formalized systems without the need for understanding the mathematical background in depth. Further, implementation would greatly benefit from a procedure that allows clinicians to formalize idiographic theories using graphical tools. Such tools could incorporate a simple three-step procedure: In a first step, clinician and patient collaboratively specify variables and sketch relations between the variables. Second, they select the qualitative nature of these specified relationships from the aforementioned list. This step encompasses the derivation of differential equations adapted to clinical practice. Third, simulations are conducted and patient and therapist can interpret and explore symptom dynamics given the case conceptualization and the differential effects of interventions.

#### Clinicians’ skepticism and utility

Recent investigations suggest that clinicians are skeptical regarding the utility of idiographic assessment approaches, specifically regarding ESM data collection and modeling techniques [24, 59]. While these surveys suggest that clinicians find idiographic data models to be generally intuitive and aligning well with their clinical reasoning, it was also found that clinicians are not always convinced that they can learn something new from idiographic data models. Further, recent studies suggest that there is little incremental information in time-series measures beyond mean levels and general variability [60] and that time-series effects show largely unacceptable reliability after partialling out redundancies with mean and variability [61]. It is important to note that these findings pertain to the utility of idiographic *data models*. As discussed above, these data models face several challenges in the clinical context (e.g., insufficient number of observations, time-scaling, measurement artifacts, modeling assumptions), offering a potential explanation for the questionable performance of time-series measures.

Formalized idiographic *theories*, on the other hand, aim to *explain* phenomena that can be observed in the patient. They do so by representing the system posited to give rise to the phenomenon. We outlined how formalizing such systems can foster theory development and therefore potentially help clinicians gaining insight into the effects of (formalized) clinical interventions. Valid inferences from such intervention simulations require clinicians to thoroughly evaluate their theories, and formalizing theories can help in doing so. We argue that, if proof-of-principle studies can support the hypothesis that formalizing idiographic theories improve treatment planning, this could greatly benefit clinical practice. However, to facilitate implementation, future research should conduct surveys with practitioners to understand potential barriers of implementing formalized idiographic theories.

#### Linear versus non-linear dynamics

We introduced two perspectives in constructing idiographic systems: First, a *top-down approach* in which generic factors are modeled and subsequently personalized through adapting parameters, and second, a *bottom-up approach* in which personalized factors are modeled directly—extending the search horizon to incorporate any factor that can be related to the patient’s system. In the present paper, we formalized a case conceptualization within the generic framework of functional analysis, using (primarily) linear equations. It is important to note that, especially when following the bottom-up approach of constructing idiographic systems for and with each patient, system dynamics should encompass not only linear, but also non-linear dynamics. Indeed, prior research examining the quality of system dynamics found that processes in therapy are often non-linear and chaotic [41, 62]. Such dynamics are, by definition, hard to predict and are heavily dependent on the specific set-up of the simulation; slight changes in the set-up of initial conditions and parameters might have dramatic effects on the simulated behavior. In such cases, it may only be possible to make broad predictions about expected behavior, for example, not when a panic attack will occur, but rather whether a system is vulnerable to such attacks. We encourage future research to further investigate how such dynamics should precisely be incorporated in the formalization of theories.

#### Incorporating social and contextual dynamics

Computational models, as the one presented in this paper, can account for processes that occur within an individual, and explain psychopathology on the basis of reinforcing factors. However, it seems unrealistic that these processes occur in isolation, independent from a social context. Indeed, clinical reasoning often includes the influence of the social environment on certain psychological processes, for instance, the link between avoidant coping tendencies and a certain attachment style, or the influence of peers in substance use. Incorporating interactions between different systems could open doors to model these clinical phenomena. Future research could use methods from agent-based modeling to simulate social interactions between patient-specific computational models and investigate how these interactions can inform parameters or variables in the patient’s system.

#### Proof-of-principle

In order for new techniques to be considered relevant to clinical practice, they should provide practitioners with a clear incentive, and a main incentive for psychotherapy is to improve treatment outcomes. For many health care systems, case conceptualizations form the starting point for hypothesis-driven intervention planning and execution. We expect that formalizing idiographic theories can improve the precision of intervention predictions, through enhancing explanatory and predictive precision in formulating case conceptualizations; however, this idea needs empirical support. We hope that future research will follow up on this hypothesis and provide us with proof-of-principle studies validating the utility of formal theories in enhancing predictive precision of case conceptualizations.

## Conclusion

Complexity models are of great relevance for psychotherapy. Case conceptualizations, even if only incorporating a small set of variables, can produce highly complex behavior. We present the formalization of idiographic theories through differential equations as an approach to align the movement of process-based psychotherapy to dynamical system methodology. Simulation results based on formalized theories can account for considerations that are vital to clinical practice. Furthermore, the process of formalizing a system promotes more scientific rigor in clinical practice and could help in improving explanatory and predictive precision of case conceptualizations, as well as treatment planning.

## Supplementary information

Additional file 1: Mathematical background. This file includes the mathematical background, including differential equations for the system variables and interventions, as well as parameter choices and initial values to conduct the simulations.

Additional file 2: Code to reproduce analyses. This file provides the code to reproduce all analyses discussed in this report in the open-source software R. All materials are made available in the open-science-framework repository: https://osf.io/spb37/.

## Abbreviations

*Sd*: Discriminant stimulus
*Cat (Rc)*: Catastrophizing (*cognitive reaction*)
*Av (Rb)*: Avoidance (*behavioral reaction*)
*Pan (Re)*: Panic (*emotional reaction*)
*Ben*: Perceived benefits of avoidance behavior
*Cost*: Perceived costs of avoidance behavior
*Cred*: Credibility of dysfunctional interpretation
*FunCog*: Credibility of functional interpretation
