# Supplementary material for: Bridging the gap between complexity science and clinical practice by formalizing idiographic theories: a computational model of functional analysis
Source: BMC Med. 2020 Apr 8;18:99. doi: 10.1186/s12916-020-01558-1 (PMC7333286; doi:10.1186/s12916-020-01558-1)
Supplement: Supplementary file 1 — Mathematical background. This file includes the mathematical background, including differential equations for the system variables and interventions, as well as parameter choices and initial values to conduct the simulations. [file 12916_2020_1558_MOESM1_ESM.pdf]

Supplemental Material: Mathematical Background

This section is meant to give an overview over the mathematical aspects of the model used in the paper. Note that the paper can be followed without having read this section, it is meant for the reader interested in some more mathematical detail.

Modeling external input: discriminant stimulus

First, we modelled input of the discriminant stimulus as a basis of panic and coping dynamics. This variable is considered *external*, meaning that it is not caused by any other variables within the individual's system. The extent to which the individual is exposed to the discriminant stimulus was defined for each time-point by a chance of 12 (not exposed) to 2 (exposed), which results in roughly a 14% chance of exposure at every time-point.

System specification: differential equations

Second, we derived first-order differential equations for all simulation studies from the schematic depiction of the system as shown in Figure 3. Generally, we modelled the momentary rate of change as a function of input variables and a decay term of the variable. The basis for the interaction between panic and avoidant coping were non-linear Lotka-Volterra dynamics, which we extended with other variables related to coping. Since we considered the perceived benefits and the credibility of the catastrophic interpretation as variables that *evaluate* the current coping outcomes, rather than being directly influenced by current levels of panic/avoidance, we included the full term of panic and avoidant coping as components of the respective equations, resulting in the following system equations:

Catastrophizing.

$$\frac{dCat}{dt} = a \cdot Sd + b \cdot Cred - c \cdot Cat \quad (1)$$

Panic.

$$\frac{dPan}{dt} = d \cdot Cat - e \cdot Pan \cdot Av - f \cdot Pan \quad (2)$$

Avoidance behavior.

$$\frac{dAv}{dt} = g \cdot Pan \cdot Av - h \cdot Cost + i \cdot Ben - j \cdot Av \tag{3}$$

Perceived benefits.

$$\frac{dBen}{dt} = k \cdot [(d \cdot Cat - e \cdot Pan \cdot Av - f \cdot Pan) \cdot Av] - l \cdot Ben \tag{4}$$

Credibility of catastrophic interpretation.

$$\frac{dCred}{dt} = m \cdot [(g \cdot Pan \cdot Av - h \cdot Cost + i \cdot Ben - j \cdot Av) \cdot Pan] - n \cdot Cred \tag{5}$$

Perceived costs.

$$\frac{dCost}{dt} = o \cdot Av - p \cdot Cost \tag{6}$$

Interventions

We formalized two commonly used CBT interventions: First, exposure was implemented through setting avoidant coping to 0. Second, cognitive reappraisal was modelled through introducing a new system variable, that captures the credibility of an alternative, functional interpretation of the discriminant stimulus. This results in the differential equation

$$\frac{dFunCog}{dt} = q \cdot Cred + r \cdot FunCog. \tag{7}$$

According to our theory, the introduction of cognitive reappraisal has an impact on catastrophizing and the credibility of the catastrophic interpretation. Therefore, we extended the differential equations of these variables with the introduction of cognitive reappraisal:

$$\frac{dCat2}{dt} = a \cdot Sd + b \cdot Cred - c \cdot Cat - s \cdot FunCog \tag{8}$$

$$\frac{dCred2}{dt} = m \cdot [(g \cdot Pan \cdot Av - h \cdot Cost + i \cdot Ben - j \cdot Av) \cdot Pan] - n \cdot Cred - t \cdot FunCog \tag{9}$$

Parameter choices and initial values

As discussed in the main text, estimating parameters in differential equations from ESM data requires that all system variables can be measured on their appropriate time-scale. Another application in computational modeling is that patient and therapist can collaboratively examine the impact of varying parameters on the patient's system. Table 1 and 2 show the parameters used to conduct the simulations. These values are based on varying system parameters until sensible behavior was resembled, given the information on our hypothetical patient.

Table 1

Parameter choices for differential equations

	**Value**	**Explanation of parameter:** ***Impact of…on… (sign)***
a	1.5	Discriminant stimulus on catastrophizing **(+)**
b	2.1	Credibility of catastrophic interpretation on catastrophizing **(+)**
c	1.65	Decay of catastrophizing **(-)**
d	2	Catastrophizing on panic **(+)**
e	0.2	Panic-avoidance interaction on panic **(-)**
f	0.5	Decay of panic **(-)**
g	0.2	Panic-avoidance interaction on avoidance **(+)**
h	0.19	Perceived costs on avoidance **(-)**
i	0.2	Perceived benefits on avoidance **(+)**
j	0.22	Decay of avoidance **(-)**
k	1	Evaluation of avoidance on perceived benefits **(+)**
l	0.02	Decay of perceived benefits **(-)**
m	0.2	Evaluation of avoidance on credibility of catastrophic interpretation **(+)**
n	0.04	Decay of credibility of catastrophic interpretation **(-)**
o	0.2	Avoidance on perceived costs **(+)**
p	0.5	Decay of perceived costs **(-)**
q	0.5	Credibility of catastrophic interpretation on functional interpretation **(+)**
r	0.02	Growth of functional interpretation **(+)**
s	0.1	Functional interpretation on catastrophizing **(-)**
t	0.3	Functional interpretation on credibility of catastrophic interpretation **(-)**

Table 2

Initial values for all system variables

**Variable**	**Initial Value**
Discriminant Stimulus *(Sd)*	0
Catastrophizing *(Cat)*	0.01
Panic *(Pan)*	0.01
Avoidance *(Av)*	0.5
Perceived benefits *(Ben)*	0.01
Perceived costs *(Cost)*	0.01
Credibility of catastrophic interpretation *(Cred)*	0.01
Credibility of functional interpretation *(FunCog)*	0
